# Catheter-induced anaphylaxis and determination of the causative catheter in a patient undergoing neuroendovascular surgery: a case report

**DOI:** 10.1186/s40981-021-00463-7

**Published:** 2021-07-31

**Authors:** Yuki Sugiyama, Kaori Numata, Natsuko Watanabe, Masatoshi Urasawa, Toru Murakami, Ikuko Murakami, Jun-Ichi Koyama, Mikito Kawamata

**Affiliations:** 1 Department of Anesthesiology and Resuscitology, Shinshu University School of Medicine, 3-1-1, Asahi, Matsumoto, Nagano 390-8621 Japan; 2 Neuroendovascular Therapy Center, Shinshu University Hospital, 3-1-1, Asahi, Matsumoto, Nagano 390-8621 Japan

**Keywords:** Anaphylaxis, Catheter, Neuroendovascular surgery, Basophil activation test, Kounis syndrome

## Abstract

**Background:**

Anaphylaxis caused by a catheter itself used for endovascular surgery is rare, and a method for detection of a causative catheter has not been established. We report a case of catheter-induced anaphylaxis in which the causative catheter was successfully detected.

**Case presentation:**

A 47-year-old male underwent neuroendovascular surgery. During surgery, blood pressure suddenly dropped and the level of tryptase indicated the occurrence of anaphylaxis. There were 24 candidate agents for the cause of anaphylaxis including 8 catheters. We performed the basophil activation test by directly mixing the catheter with blood. One catheter coated with a hyaluronic acid product showed a positive reaction, and we confirmed the result by a modified skin test using an elution solution of the catheter. Later, we successfully completed the neuroendovascular surgery without the catheter.

**Conclusions:**

The methods used in this case can be useful for the detection of the causative agent in catheter-induced anaphylaxis.

## Background

Although catheter surgery has been performed in various medical fields, anaphylaxis caused by the catheter itself is rare. Once anaphylaxis occurs, detection of the causative agent is important; however, identification of the agent is difficult since many candidates for drugs and medical equipment are used during catheter surgery. In case in which a catheter is the candidate, its detection is challenging because a detection method has not yet been established. We report a case of catheter-induced anaphylaxis during neuroendovascular surgery in which the causative catheter was successfully detected by a modification of the standard basophil activation test (BAT) [1] and was confirmed by a modification of the skin test. Written informed consent was obtained from the patient for publication of this case report.

## Case presentation

A 47-year-old male (height, 175 cm; weight, 99 kg) was scheduled to undergo neuroendovascular surgery for a right middle cerebral artery aneurysm. Cerebral angiography was performed 3 months ago, and neuroendovascular surgery was indicated because there were many perforator arteries around the aneurysm and surgical clipping was considered to be difficult. He was receiving oral treatment for diabetes and hypertension, and he had no history of allergies.

General anesthesia was induced with 140 mg of propofol, 100 μg of fentanyl, 0.1 μg/kg/min of remifentanil, and 60 mg of rocuronium. After tracheal intubation, general anesthesia was maintained with 1.5% of sevoflurane and 0.1 μg/kg/min of remifentanil. Three μg/kg/min of dopamine, 1 g of cefazoline, and 3.3 mg of dexamethasone for a prophylactic antiemetic, iohexol, and heparin were administered, and the surgery proceeded uneventfully for about 1 h.

**Fig. 1:**
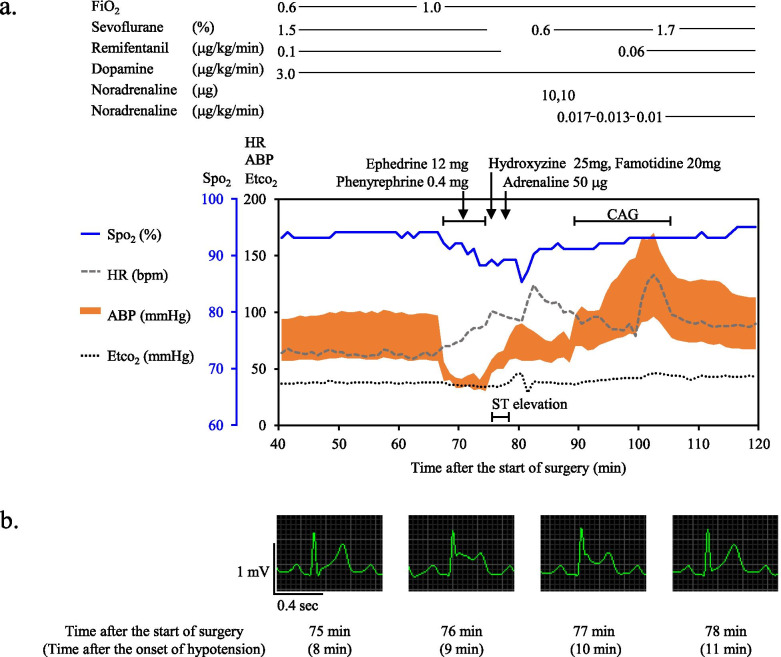
Clinical course and identification of the causative agent of anaphylaxis. **a** Anesthetic chart during neuroendovascular surgery. **b** ST segment elevation after onset of hypotension. FiO_2_ fraction of inspiratory oxygen, SpO_2_ percutaneous oxygen saturation, HR heart rate, bpm beats per minute, ABP arterial blood pressure, EtcO_2_ endo-tidal carbon dioxide, CAG coronary angiography

A guiding catheter was uneventfully placed at the right carotid artery, and a hydrophilic microcatheter (length, 150 cm; diameter, 0.56–0.80 mm) (Headway 17 Advanced™, TERUMO, Tokyo, Japan) was advanced into the aneurysm. Three minutes after its placement, the arterial blood pressure (ABP) suddenly dropped from 97/57 mmHg to 56/39 mmHg and the heart rate (HR) increased from 61 beats per minute (bpm) to 73 bpm (Fig. 1a). At that time, catheter manipulation was not performed in the patient’s body. Although 12 mg of ephedrine and 0.4 mg of phenylephrine were administered within 8 min, they were ineffective and percutaneous oxygen saturation (SpO_2_) gradually worsened to 89% with 100% oxygen administration. Although skin rashes and wheeze were not present, anaphylaxis was suspected and 25 mg of hydroxyzine, 20 mg of famotidine, and 50 μg of adrenaline were administered. ABP slightly recovered, but ST segment elevation in lead II occurred (Fig. 1b). The microcatheter and guiding catheter were removed, and 3 mg/h of nicorandil and 0.017 μg/kg/min of noradrenaline were administered. ST elevation returned to the baseline within 3 min, and ABP recovered to 98/50 mmHg and HR was 90 bpm. Skin symptoms did not appear even after recovery of blood pressure. Emergency coronary angiography was immediately performed using an arterial sheath placed for neuroendovascular surgery and revealed no significant coronary stenosis. The levels of serum tryptase, which were later obtained, were 73.8 μg/L, 34.1 μg/L, and 6.8 μg/L at 30 min (acute phase), 2 h (acute phase), and 24 h (baseline) after the onset of hypotension, respectively. The levels of serum tryptase in the acute phase were greater than [(1.2 × baseline tryptase level) + 2] μg/L [2], and we made a diagnosis of anaphylaxis. ST elevation was considered to be due to hypotension or coronary spasm known as Kounis syndrome [3]. The surgery was discontinued, and the patient was transferred to the intensive care unit. The patient gradually recovered and was extubated on the day of surgery. The patient was discharged, and allergy tests were performed 6 weeks later.

A total of 24 agents including 7 drugs, 7 kinds of medical equipment such as a urinary catheter and antiseptics, 8 kinds of catheters for neuroendovascular surgery, and 2 accessories of catheters were used during surgery (Table 1). Information on characteristics of each of the 8 catheters was obtained from the manufacturers as personal communications. Although ethylene oxide gas was used for sterilization of all catheters, a specific IgE measurement for ethylene oxide gas (Thermo Fisher Diagnostics K.K., Tokyo, Japan) was negative. In order to avoid patient burden, we firstly performed the BAT using Allergenicity Kit™ (Beckman Coulter, Marseille, France) with an additional anti-CD63-APC antibody. Although a method of the BAT for a catheter had not been established, we performed it by directly mixing the catheter itself with blood in vitro instead of mixing the solution of the coating material. Briefly, 100 μl of EDTA-collected whole blood was incubated with 100 μl of activation solution, 20 μl of CRTH2-FITC/CD203c-PE/CD3-PC7 reagent, 0.5 μl of anti-CD63-APC antibody, and 20 μl of allergen for 15 min at 37ºC. For the catheter, each sample was cut into lengths of 1 cm, and two parts were put into a 1.5-ml microtube with 20 μl of phosphate-buffered saline instead of 20 μl of allergen. Basophil activation was determined by CD203c and CD63 upregulation in a FACS Canto II flow cytometer (Becton Dickinson, San Jose, CA, USA). The result is considered positive when the percentage of activated basophils after stimulation with allergens is > 5% and the stimulation index (ratio of the percentage of activated basophils after stimulation to the percentage of activated basophils in negative control) is > 3 [4].

**Table 1:** Drugs and materials of medical equipment used during neuroendovascular surgery and previous diagnostic angiography

**Kinds of medical equipment (Number)**	**Agents and coating materials**
**Drugs (7)**	
1.	Propofol
2.	Fentanyl
3.	Remifentanil
4.	Rocuronium
5.	Cefazolin
6.	Iohexol
7.	Heparin
**Medical equipment other than catheters (7)**	
1. Urinary catheter kit	Urinary catheter
2.	Glove
3.	Jerry
4.	Benzalkonium chloride
5.	Olanexidine gluconate
6. Antiseptic	Chlorhexidine
7. Glove	Latex
**Catheters and their accessories during surgery (10)**	
1. Guiding catheter	Maleic anhydride
2. Stiff wire guide	Polytetrafluoroethylene
3. Guide wire	Polyvinyl pyrrolidone
4. Sheath kit (sheath)	Maleic anhydride
5. Sheath kit (accessory 1)	Maleic anhydride
6. Sheath kit (accessory 2)	Non-coating
7. Guiding catheter	Maleic anhydride
8. Guide wire	Polyvinyl pyrrolidone
9. Microcatheter	Hyaluronic acid product
10. Microcatheter	Polyacrylamide/polyvinyl pyrrolidone
**Catheters used during previous diagnostic angiography (3)**	
1. Guide wire	Maleic anhydride
2. Sheath kit	Non-coating
3. Guiding catheter	Maleic anhydride

**Fig. 2:**
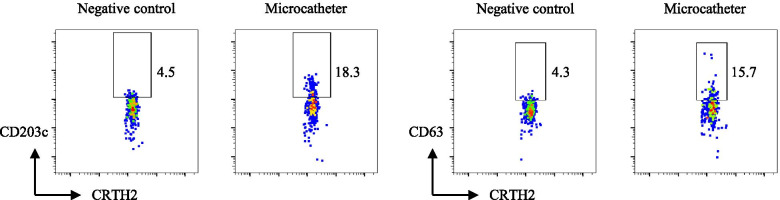
Basophil activation test. The basophil population (side scatter low CD3 − CRTH2 + cells) is shown, and the numbers represent the percentages of activated basophils

The BAT was performed in all of the 24 possible causative agents used during surgery and showed a positive reaction in only the microcatheter (Headway 17 advanced™), which was inserted about 3 min before the onset of anaphylaxis (Fig. 2). To confirm this result and to assess the possibility that the patient was sensitized by catheters used in previous diagnostic angiography, we tried to perform a skin test for the microcatheter and 3 kinds of catheters previously used. As the allergen of each catheter, we made an elution solution of the coating material by immersing the catheter itself in normal saline. Briefly, each catheter was cut into lengths of 1 cm, and two parts were put into a 1.5-ml microtube with 1 ml of normal saline. The tube was incubated at 37℃ for 1 h, and the solution was used as the allergen. In accordance with results of the BAT, only the microcatheter was positive in the intradermal test. The causative microcatheter was coated with a hyaluronic acid product, and other catheters were coated with various kinds of synthetic polymer compounds (Table 1). We additionally performed the BAT and skin test of the causative microcatheter coated with a hyaluronic acid product in 3 healthy volunteers who were not exposed to the catheter and did not have a history of any allergies to investigate whether the positive reactions in the BAT and skin test of the catheter were non-specific reactions. The study protocol was set in the same way as that of the patient’s protocol and was approved by the ethical committee of our institution (No.5192). Written informed consent was obtained from each volunteer. The results of the tests were negative in all of the healthy volunteers, and we confirmed that the positive reaction in the patient was true.

Five months after the surgery, we successfully completed the second neuroendovascular surgery for the patient with catheters that were not coated with a hyaluronic acid product.

## Discussion

There are many possible causes of sudden onset of hypotension during neuroendovascular surgery including vagal reflex or coronary artery spasm due to catheter manipulation, catheter-induced vascular perforation, rupture of an aneurysm, and anaphylaxis. In our case, although skin rashes were not present, since vasopressor-resistant hypotension was accompanied by exacerbation of oxygenation and since surgery proceeded uneventfully and catheter manipulation was not performed just before the onset of hypotension, we suspected the onset of anaphylaxis. Transient ST elevation was considered to be induced by hypotension or coronary spasm [3, 5, 6]. ST segment resolution was observed immediately after administration of antihistaminergic agents and adrenaline, suggesting that these drugs were effective against coronary spasm and/or vasodilation induced by anaphylaxis.

The occurrence of anaphylaxis was later confirmed by elevation of the tryptase level, and we tried to identify the causative agent and select safe catheters for a reoperation in the patient, because surgical clipping was predicted to be difficult and endovascular surgery was required. We firstly performed the BAT with a modified method to avoid the patient’s burden of skin tests with many kinds of agents. We successfully detected the causative catheter and confirmed the result by a modified skin test.

To the best of our knowledge, this is the first case of anaphylaxis induced by a catheter itself used for catheter surgery. In addition, this is the first case in which the causative catheter of anaphylaxis was determined by the BAT. Previously reported causes of catheter-related anaphylaxis were latex [7], ethylene oxide gas [8], chlorhexidine [9], and agents for treatment [10] used as coating agents of central/peripheral vein catheters [11, 12], urinary catheter, or intrauterine pressure catheter [13]. Unlike those coating agents, coating materials for improving lubricity or hydrophilicity in catheter treatment as shown in Table 1 are unfamiliar for us and are difficult to obtain.

The BAT has the advantage of the basophil being able to react with these candidate materials by directly mixing the catheter itself with blood in vitro, but successful detection by this method for catheter-induced anaphylaxis has not been reported. The causative agent was strongly suspected to be an agent of hyaluronic acid derivatives, which has not previously been reported as the cause of anaphylaxis. We could not determine its structural formula because there are many hyaluronic acid products used in clinical practice that have molecular weights ranging from several kilodaltons to thousands of kilodaltons. Since many kinds of hyaluronic acid derivatives are widely used in daily life, this patient might have been sensitized by some kind of hyaluronic acid product before the first neuroendovascular surgery.

## Conclusions

We presented a case of catheter-induced severe anaphylaxis during neuroendovascular surgery. Since new catheters and materials are expected to increase, the incidence of catheter-related anaphylaxis may increase. The methods used in this case can be useful for the detection of the causative agent in catheter-induced anaphylaxis.

## Data Availability

Not applicable due to patient privacy concerns.

## Abbreviations

ABP: Arterial blood pressure
BAT: Basophil activation test
bpm: Beats per minute
CAG: Coronary angiography
EtcO_2_: Endo-tidal carbon dioxide
FiO_2_: Fraction of inspiratory oxygen
HR: Heart rate
SpO_2_: Percutaneous oxygen saturation
